# Persisting disparities in hepatitis B diagnoses, associated risk factors, and time to diagnosis among migrants compared with Danish-born populations: a matched cohort study

**DOI:** 10.1093/eurpub/ckag167

**Published:** 2026-09-14

**Authors:** Anna Deal, Farah Seedat, Jessica Carter, Kristina Langholz Kristensen, Sally E Hayward, Kenneth Kabagambe, Jørgen Holm Petersen, Nina Weis, Philippa Matthews, Maria Wessman, Jon S Friedland, Sally Hargreaves, Marie Norredam

**Affiliations:** The Migrant Health Research Group, School for Health and Medical Sciences, City St. George’s, University of London, London, United Kingdom; Faculty of Public Health and Policy, London School of Hygiene and Tropical Medicine, London, United Kingdom; The Migrant Health Research Group, School for Health and Medical Sciences, City St. George’s, University of London, London, United Kingdom; The Migrant Health Research Group, School for Health and Medical Sciences, City St. George’s, University of London, London, United Kingdom; Wolfson Institute of Population Health, Queen Mary, University of London, London, United Kingdom; Department of Public Health, Danish Research Centre for Migration, Ethnicity and Health, Section of Health Services Research, University of Copenhagen, Copenhagen, Denmark; Department of Pulmonary and Infectious Diseases, Nordsjaellands Hospital, Hilleroed, Denmark; The Migrant Health Research Group, School for Health and Medical Sciences, City St. George’s, University of London, London, United Kingdom; National Organization for People Living with Hepatitis B, Kampala, Uganda; Department of Public Health, Danish Research Centre for Migration, Ethnicity and Health, Section of Health Services Research, University of Copenhagen, Copenhagen, Denmark; Department of Clinical Medicine, Faculty of Health and Medical Sciences, University of Copenhagen, Copenhagen, Denmark; Department of Infectious Diseases, Hvidovre University Hospital, Hvidovre, Denmark; The Francis Crick Institute, University College London, London, United Kingdom; Division of Infection and Immunity, University College London, London, United Kingdom; Department of Infectious Diseases, University College London Hospital, London, United Kingdom; Infectious Disease Epidemiology and Prevention/Sex and Blood Transmitted Infections, Statens Serum Institut, Copenhagen, Denmark; The Migrant Health Research Group, School for Health and Medical Sciences, City St. George’s, University of London, London, United Kingdom; The Migrant Health Research Group, School for Health and Medical Sciences, City St. George’s, University of London, London, United Kingdom; Department of Public Health, Danish Research Centre for Migration, Ethnicity and Health, Section of Health Services Research, University of Copenhagen, Copenhagen, Denmark; Department of Infectious Diseases, Hvidovre University Hospital, Hvidovre, Denmark

## Abstract

Hepatitis B virus (HBV) remains a major public health concern globally despite the availability of an effective vaccine. In Europe, HBV risk is particularly high among those originating from areas with high prevalence rates. We investigated diagnosis rates and risk factors for HBV infections among migrants compared to Danish-born populations. Our matched cohort study included migrants matched 1:6 with Danish-born individuals over a 23-year period. We calculated HBV diagnosis rates per 10 000 person-years. A multivariate quasi-Poisson regression analysis was done across the entire cohort, to compare adjusted incidence rate ratios (aIRRs) between the Danish-born and migrant cohorts. A further multivariate analysis was done in the migrant cohort only, to examine factors associated with diagnosis. Time to diagnosis was also compared within the migrant cohort. 179 110 migrants and 1 049 345 Danish-born individuals were included in the study cohort, among whom there were 1362 HBV diagnoses (migrants: 1118; Danish-born: 244). Migrants had a higher HBV diagnosis rate compared to Danish-born (aIRR 30.6; 95% CI: 26.7–35.2). A higher rate of substance use disorders, hepatitis C, and HIV co-infection were observed among Danish-born cases. Within the migrant cohort, hepatitis C diagnosis, region of origin (Southeast Asia and Pacific, African region), and migrant status were associated with HBV diagnosis and the average time from arrival to diagnosis was 5.4 years. Migrants are at increased risk for HBV and may experience diagnostic delays, particularly those who fall outside of routine screening processes for pregnant women and on-arrival health assessments. At-risk migrant groups should be engaged early in culturally competent screening programs to increase health equity and meet WHO 2030 HBV elimination goals.

## Introduction

Chronic hepatitis B (HBV) remains a major public health concern globally, despite the availability of a number of highly effective vaccines. In 2022, the World Health Organization (WHO) estimated that 254 million people were living with chronic HBV infection worldwide, and an estimated 1.1 million deaths due to HBV in the same year [1, 2]. WHO targets from their Global Health Sector Strategy on HIV, Viral Hepatitis, and Sexually Transmitted Infections (2022–30) aim to eliminate viral hepatitis as a public-health threat by 2030, targeting a 90% reduction in new infections and 65% reduction in related mortality [3]. Achieving these elimination goals in low prevalence European countries will require addressing persistent disparities in HBV burden, particularly among migrants originating from high prevalence areas [4, 5].

In the WHO European Region, most new diagnoses of HBV are among people born outside the host country [4, 5]. In a recent systematic review, it was found that of all (*n* = 53) WHO European Region countries, only 3 (6%; France, Italy, UK) had published guidelines or policies around screening migrants or refugees for HBV, and only 2 (4%; Italy, UK) countries were aligned with WHO/ECDC guidelines [6]. In Denmark, although the overall prevalence of chronic HBV infection is estimated to be below 0.3% [7], prevalence among specific migrant groups is estimated to be higher, reflecting prevalence in some countries of origin [8]. HBV is a notifiable disease in Denmark, with mandatory reporting of acute cases since 1980 and chronic infections since 2000. Universal screening of pregnant women was introduced in 2005, but other at-risk groups are screened inconsistently. Asylum seekers and quota refugees [refugees resettled in Denmark following an agreement with the United Nations High Commissioner for Refugees (UNHCR) or a similar international organization [9]] may receive a general health assessment (GHA) that may include HBV testing, but implementation levels are unclear in these groups [10]. Previous studies suggest that up to 7% of HBV infections in Denmark remain undiagnosed and that fewer than one-third of diagnosed individuals are linked to specialist care [7]. Migrants in Denmark, and Europe more widely, account for a disproportionate share of HBV cases, yet are generally known to face more barriers to diagnosis, treatment, and follow-up than the general population, which may lead to slower diagnosis and poorer outcomes in this group [4, 5, 7]. These gaps may contribute to the ongoing existence of HBV in Denmark and prevent it, alongside other European countries, from achieving WHO 2030 elimination targets.

Despite the strong national surveillance system in Denmark, there is currently no published evidence on the extent to which diagnosis rates differ between migrants and Danish-born, or if risk factors associated with HBV vary between these two population groups. Earlier research has focused mainly on prevalence or clinical cohorts [7]. This gap is critical because understanding these differences is essential for designing appropriate, evidence-based prevention strategies. Therefore, we used Danish registry data to compare HBV diagnosis rates in migrants and Danish-born individuals between 1993 and 2015. Using a matched cohort design, we explored the influence of demographic and clinical factors on HBV risk. Within the migrant cohort, we further analyzed differences by region of origin and legal migration status and estimated time from arrival in Denmark to HBV diagnosis. By characterizing HBV incidence using Denmark’s comprehensive and nationwide register-based data, this study aims to inform equitable, evidence-based screening and prevention strategies that may hold relevance in Denmark and similar low-endemic, migrant-receiving countries, contributing toward meeting the WHO 2030 HBV elimination goals.

## Methods

### Study design, outcomes, and exposures

We completed a retrospective register-based, matched cohort study using database linkage to obtain the exposure and outcome variables. Our primary exposure variable was whether the participant was a migrant or Danish-born individual. Our primary outcome variable was diagnosis of HBV diagnosis, acute or chronic. Our secondary outcomes were clinical outcomes related to HBV, namely hospitalization for acute HBV, acute HBV with coma, and liver cancer, liver cirrhosis, or liver failure. Several secondary exposure variables were included as potentially confounding variables when (i) comparing the rate of diagnosis of HBV in migrants compared with Danish-born individuals and (ii) assessing the risk factors associated with HBV in migrant populations. These were: age, sex, comorbidities (hepatitis C, HIV, and substance use disorder), migrant status, and area of origin. Migrant status was broken up into three groups: Asylum-pathway refugees (granted refugee status after seeking asylum), Quota refugees (resettled in Denmark following an agreement with the United Nations High Commissioner for Refugees (UNHCR) or a similar international organization [9]), and family-reunified to either Danish/Nordic citizens, immigrants, or refugees (through family reunification visas). Migrants on other visas, such as work permits, or undocumented migrants were not included. Area of origin was divided into six groups, due to the structure of the existing cohort (described further by [11]): Eastern Europe and Central Asia; Europe, North America and Oceania (“Western”); Middle East and North Africa; Latin America and the Caribbean; South and South-East Asia and Pacific (SSEA and Pacific); and sub-Saharan Africa. Countries included in each region are listed in Supplementary Table S1.

### Data sources and participants

We used data from the Danish Migrant Cohort, an existing register-based cohort of migrants (refugees and family-reunified migrants) obtaining a residence permit in Denmark between 1 January 1993 and 31 December 2015 (end date of the Danish Migrant Cohort). In this cohort, each included migrant was matched to six Danish-born individuals identified through Statistics Denmark based on age and sex. All Danish-born individuals are assigned a unique personal identification (PIN) number at birth, whereas migrants are registered when resettling after obtaining residency. The PIN can be used to track individuals through public registries at an individual level. We included migrants and their matched individuals from the date of receiving residency. The construction of the cohort has previously been described in more detail including the matching procedure and exclusion of missing values [12, 13].

We obtained data on our primary outcome variable of HBV diagnosis by cross-linking migrants and their matches through their CPR numbers to records of HBV diagnoses held in the National Surveillance Register (NSR), Department of Infectious Disease Epidemiology and Prevention (DIDE) at the Statens Serum Institut (SSI). Hepatitis B is a notifiable disease by law and diagnosing doctors are responsible for reporting the disease in writing to the SSI. Reporting newly diagnosed acute HBV to the register has been mandatory since 1980 and notification of chronic HBV infection became mandatory in the year 2000 [7]. In addition to this outcome variable, we also obtained data on HIV diagnoses (HIV is also notifiable in Denmark) as additional exposure variables from the NSR, DIDE. As an individual can only be infected with hepatitis B once in their lifetime, migrants with a recorded diagnosis of HBV before the date of residency (*n* = 72) were excluded from further analysis, along with Danish born individuals diagnosed before study start (*n* = 482, including *n* = 242 diagnosed between 1993 and the individual’s entry into the study cohort) and all matches of excluded individuals.

Finally, we obtained data on hospital-based (both inpatient and outpatient) cases of substance use disorders, hepatitis C, and the remaining clinical outcomes of hospitalization for acute HBV, acute HBV with coma, and liver cancer, liver cirrhosis, or liver failure by cross-linking the PIN numbers of migrants and their matches to records from hospital registers.

### Statistical analysis

We described the categorical exposure and outcome variables using frequencies and percentages and the continuous exposure and outcome variables using means and standard deviations and medians with interquartile ranges. We calculated rate of diagnosis of HBV per 10 000 person-years. We then calculated unadjusted diagnosis rate ratios [referred to from this point onwards as incidence rates ratios (IRR), as per statistical convention] and adjusted (aIRRs) using quasi-Poisson regressions to account for under-dispersion. We adjusted the aIRRs for sex, age, substance use disorder, HIV, and hepatitis C comorbidities. We also calculated diagnosis rates per 10 000 people for every year between 1994 and 2015 to explore the trends of HBV across time.

Finally, we explored factors associated with HBV in migrant populations also by calculating the IRR and aIRR in the migrant population only using quasi-Poisson regressions, including all the factors (age, sex, substance use disorder, HIV, hepatitis C) in addition to area of origin and migrant status.

All data cleaning and analyses were carried out using R version R v4.2.1.

### Ethical approval

This study was approved by the Danish Data Protection Agency (number 2016-41-4576). No further ethical approval is required in Denmark for registry-based research.

## Results

### Cohort demographics

In total, 1 228 455 individuals were included in the cohort, of whom 1 049 345 were Danish-born and 179 110 were migrants, as shown in Supplementary Table S2. In the cohort overall, 45.9% of individuals were male and 54.1% female. A large majority of the cohort was born between 1950 and 1990 (81.8%), with the average age at study start (an individual’s entry into the cohort) being 28.2 years (SD: 13.3 years). The proportion of individuals affected by co-morbidities varied between the migrant and Danish-born population, with HIV and hepatitis C more prevalent in the migrant cohort and substance use disorder more prevalent in the Danish-born cohort.

In the migrant cohort, the most common areas of origin were the Middle East and North African region (28.3%), the Central Asia and Eastern European region (24.9%), and the Southeast Asia and Pacific Region (20.4%). Almost half (46.8%) of the migrant cohort were asylum-pathway refugees, with most of the remainder of the cohort (33.1%) being family reunified to Danish citizens.

### Characteristics of HBV cases and clinical outcomes

Across the entire cohort, there were a total of 1362 new HBV diagnoses within the study period. Of these, 1118 (82.1%) were in the migrant cohort and 244 (17.9%) in the Danish-born cohort (Table 1). In the Danish-born cohort, there were more diagnoses among males (54.9%) than among females (45.1%), whereas this was reversed in the migrant cohort, among whom more diagnoses were in women (61.7%). Overall, among both migrant and Danish-born women (*n* = 800), 92.6% of HBV diagnoses were among those of child-bearing age (18–49). Among the migrant cohort, the majority (54.2%) of diagnoses were within 5 years of their arrival in Denmark.

**Table 1:** HBV diagnoses and their demographic and clinical characteristics by migrant status during the study period.

	**Danish-born (*n* = **1 049 345)	**Migrant (*n* = **179 110)	**Total(*n* = **1 228 455**)**
HBV diagnoses, *N*	244	1118	1362
Sex, *N* (%)			
Male	134 (54.9)	428 (38.3)	562 (41.3)
Female	110 (45.1)	690 (61.7)	800 (58.7)
Women of child-bearing age (18–49)	104 (42.6)	637 (56.9)	741 (54.4)
Mean year of diagnosis (IQR)	2006 (2002–10)	2008 (2004–12)	2007 (2003–11)
1993–99	34 (13.9)	59 (5.3)	93 (6.8)
2000–05	87 (35.7)	300 (26.8)	387 (28.4)
2006–10	64 (26.2)	333 (29.8)	397 (29.1)
2011–15	59 (24.2)	426 (38.1)	485 (35.6)
Mean age at diagnosis (SD)	33.7 (10.6)	34.1 (10.3)	34.0 (10.4)
<18 years	9 (3.7)	46 (4.1)	55 (4.0)
18–30 years	86 (35.2)	361 (32.2)	369 (27.1)
31–40 years	97 (39.8)	452 (40.4)	549 (40.3)
>40 years	52 (21.3)	259 (23.2)	311 (22.8)
Risk factors/comorbidities 1993–2015, *N* (%)			
HIV+	6 (2.5)	7 (0.6)	13 (1.0)
Substance abuse	51 (20.9)	17 (1.5)	68 (5.0)
Hepatitis C+	36 (14.8)	17 (1.5)	53 (3.9)
Mean time between arrival in Denmark/study start to diagnosis (SD)	6.9 (5.0)	5.8 (5.4)	
0–5 years	102 (41.8)	606 (54.2)	
5–10 years	81 (33.2)	249 (22.2)	
>10 years	61 (25)	263 (23.5)	
Clinical outcomes, *N* (%)			
Hospitalization for acute HBV	73 (29.9)	66 (5.9)	139 (10.2)
Acute HBV with coma	^a^	^a^	7 (0.5)
Liver cancer	^a^	^a^	5 (0.4)
Liver cirrhosis or liver failure	^a^	^a^	5 (0.4)

a Small numbers suppressed for data protections purposes.

HBV, hepatitis B; HIV, human immunodeficiency virus; IQR, interquartile range; *N*, number of participants; SD, standard deviation.

The average age at HBV diagnosis was similar among migrants (34.1 years, SD: 10.3) and Danish-born (33.7 years, SD: 10.6), with the age group of 31–40 years being the most commonly diagnosed in both cohorts. By contrast, the pattern of co-morbidities varied across the cohorts, with HBV diagnoses among Danish-born being more frequently among those with either HIV (2.5%), hepatitis C (14.8%), or substance use disorder (20.9%), compared with those in the migrant cohort. In the migrant cohort, only 1.5% of HBV diagnoses were among those with hepatitis C or a substance use disorder, and 0.6% among those with HIV.

In terms of clinical outcomes, almost one-third (29.9%) of cases in the Danish-born cohort were hospitalized within the study period for acute HBV, compared to only 5.9% of the migrant cohort. Overall, very few individuals with a HBV diagnosis were hospitalized for a HBV-associated coma (0.5%), liver cancer (0.4%), or liver cirrhosis/liver failure (0.4%), with similar outcome patterns observed between the migrant and Danish-born cohorts.

### Rate of HBV diagnosis over time

**Figure 1:**
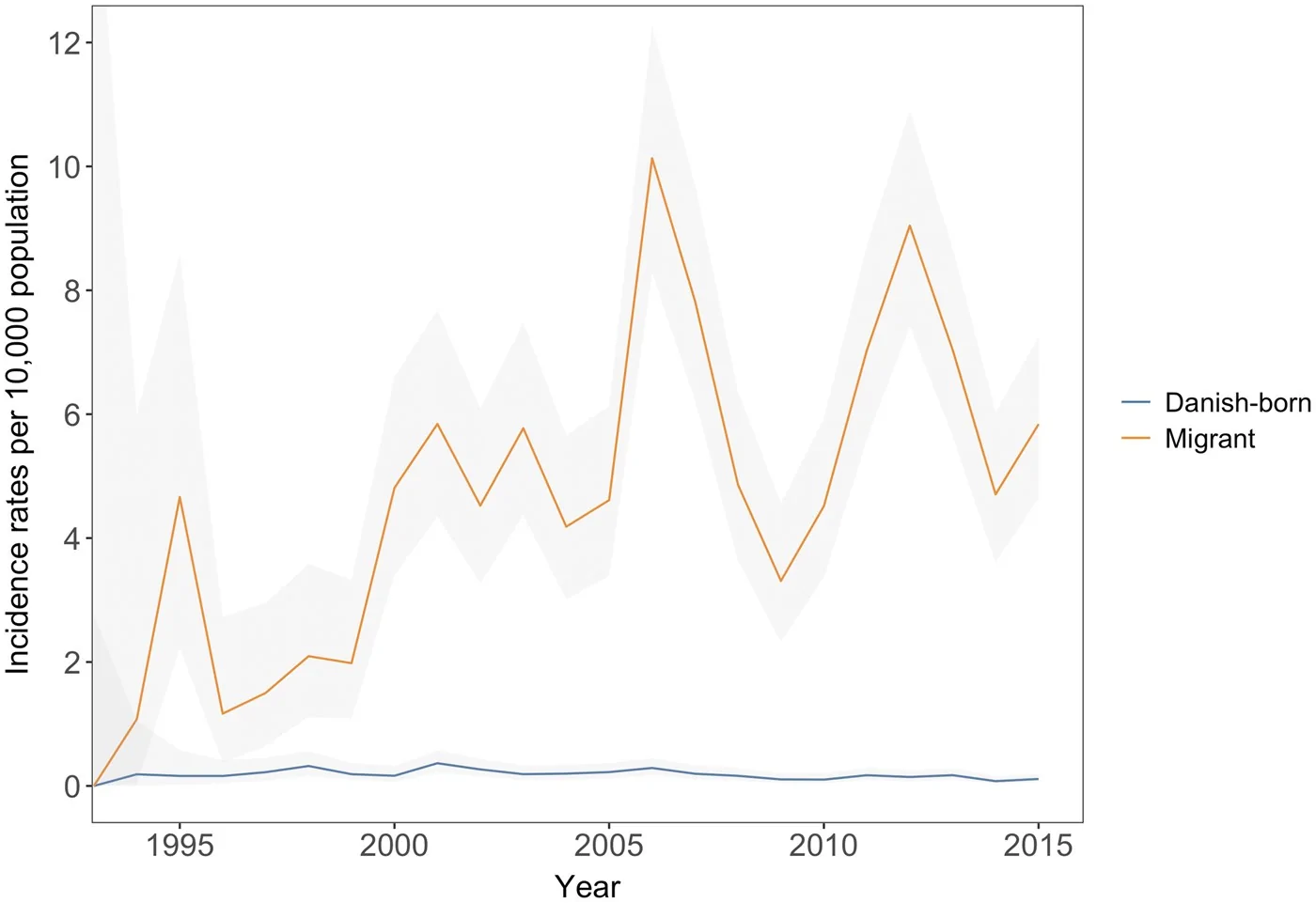
Diagnosis rates of HBV diagnoses across time in Danish-born and migrant cohorts, 1994–2015.

Across the entire study period, the average rate of new diagnoses among migrants was 5.57 per 10 000 person years (95% CI: 5.25–5.91), compared with 0.18 (95% CI: 0.16–0.21) in the Danish-born cohort. Figure 1 demonstrates an overall increase in diagnoses rates among the migrant cohort across the study period, with diagnoses among the Danish-born cohort staying steady and relatively low. In 1994 (number of individuals included in the study in 1993 was too low to generate a reliable estimate), the rate of new HBV diagnoses among migrants was 1.08 (95% CI: 0.02–6.00), compared with 5.84 (95% CI: 4.65–7.24) by 2015. Two major peaks in diagnoses among migrants were observed in 2006 (10.13; 95% CI: 8.29–12.3) and 2012 (9.04; 95% CI: 7.43–10.91). Among the Danish-born cohort, diagnoses did not go above a peak of 0.37 per 10 000 person years (95% CI: 0.22–0.57) in 2001.

### Risk factors associated with HBV diagnosis

In the study period, we found that the migrant cohort had a diagnosis rate of HBV over 30 times that of the Danish-born population, both when adjusted for other risk factors (aIRR: 30.7, 95% CI: 26.65–35.21) and alone (IRR: 30.18, 95% CI: 26.32–34.73), as shown in Table 2. In the cohort overall, being female was associated with a slightly higher diagnosis rate than being male (aIRR: 1.22, 95% CI: 1.09–1.36), but there was no association between age at study entry and HBV diagnosis rate.

**Table 2:** Unadjusted and adjusted incidence rate ratios for HBV by migration status and other demographic and clinical risk factors using quasi-Poisson regression.

	IRR (95% CI)	Adjusted IRR (95% CI)
Migrant status		
Danish-born	Ref	Ref
Migrant	30.18 (26.32–34.73)	30.7 (26.65–35.21)
Sex		
Male	Ref	Ref
Female	1.17 (1.05–1.30)	1.22 (1.09–1.36)
Age at study entry	1.00 (0.99–1.01)	1.00 (0.99–1.01)
Substance abuse		
No	Ref	Ref
Yes	1.94 (1.51–2.46)	2.87 (2.22–3.66)
HIV		
No	Ref	Ref
Yes	14.10 (7.74–23.27)	3.24 (1.77–5.39)
Hepatitis C		
No	Ref	Ref
Yes	29.05 (21.81–37.81)	16.32 (12.16–21.42)

CI, confidence intervals; HBV, hepatitis B; HIV, human immunodeficiency virus.

All three included co-morbidities were associated with higher diagnosis rates of HBV. This relationship was the most prominent among those diagnosed with hepatitis C, who had an incidence rate of HBV over 16 × (aIRR: 16.32, 95% CI: 12.16–21.42) that of those without hepatitis C (when adjusted for other risk factors). Both those diagnosed with HIV and those with a substance use disorder had a HBV diagnosis rate of around 3 × (HIV aIRR: 3.24, 95% CI: 1.77–5.39; substance abuse aIRR: 2.87, 95% CI: 2.22–3.66) that of those without each risk factor, after adjustment.

Table 3 shows the risk factors in the migrant cohort. Among the three included co-morbidities, only a hepatitis C diagnosis was associated with a higher incidence rate of HBV (aIRR: 5.90, 95% CI: 3.50–9.23), after adjustment. Age at study entry and sex were also not associated with differing HBV incidence rates after adjustment. We found that diagnosis rates in those originating from the Southeast Asia and Pacific (11.55 per 10 000 person-years, 95% CI: 10.50–12.68) and African (13.19, 95% CI: 11.82–14.67) regions were higher than the average in the migrant cohort, and when compared with those originating from Central Asia and Eastern Europe, they had three (aIRR: 3.08, 95% CI: 2.58–3.69) and 4 × (aIRR: 4.30 95% CI: 3.58–5.18) higher HBV incidence rates, respectively. In contrast, those from the Latin American and Caribbean, Middle East and North African and Western regions had significantly lower HBV incidence rates than those in the reference (Central Asia and Eastern Europe) region. Across all regions of origin, those from the Central Asian and Eastern European region had the longest average time from arrival in Denmark to diagnosis (9 years) and those from the Southeast Asian and Pacific region the shortest (3.8 years), discounting the “stateless” group. In terms of migrant status, diagnosis rates were highest among quota refugees (10.06, 95% CI: 8.24–12.15) and lowest among asylum-pathway refugees (3.47, 95% CI: 3.11–3.86), with this pattern reflected in the aIRRs (Table 3), where all other migrant status groups had significantly higher aIRRs compared to asylum-pathway refugees. The time between arrival in Denmark and HBV diagnosis was longest in those re-unified to immigrants (7.6 years) and asylum-pathway refugees (7.3 years) and lowest among quota refugees (2.4 years).

**Table 3:** Unadjusted and adjusted incidence rate ratios for HBV diagnosis by demographic and clinical risk factors in migrant populations using quasi-Poisson regression.

	*N* in cohort	Hep B cases, *N*	Mean years in Denmark at diagnosis	Incidence rates 1993–2015 per 10 000 population (95% CI)	IRR (95% CI)	Adjusted IRR (95% CI)
Overall migrant cohort	1 79 110	1118	5.8	5.57 (5.25–5.91)	–	–
Area of origin						
Central Asia and Eastern Europe	44 602	199	9	3.02 (2.61–3.46)	Ref	Ref
Latin America & Caribbean	5165	6	4.8	1.34 (0.49–2.92)	0.44 (0.18–0.92)	0.33 (0.13–0.68)
Middle East & North Africa	50 602	95	5.8	1.92 (1.55–2.34)	0.64 (0.50–0.81)	0.63 (0.49–0.80)
Southeast Asia & Pacific	36 468	441	3.8	11.55 (10.50–12.68)	3.83 (3.24–4.54)	3.08 (2.58–3.69)
Stateless	1115	^a^	3.3	^a^	1.90 (0.47–5.00)	1.74 (0.43–4.59)
African region	25 521	338	5.4	13.19 (11.82–14.67)	4.37 (3.68–5.22)	4.30 (3.58–5.18)
Western	15 637	36	5.2	2.18 (1.53–3.02)	0.72 (0.50–1.02)	0.53 (0.36–0.76)
Migrant status						
Asylum-pathway refuges	83 765	343	7.3	3.47 (3.11–3.86)	Ref	Ref
Quota Refugee	10 082	107	2.4	10.06 (8.24–12.15)	2.90 (2.32–3.58)	2.15 (1.71–2.67)
Reunified to immigrants	10 254	120	7.6	8.80 (7.30–10.52)	2.54 (2.05–3.11)	2.74 (2.20–3.38)
Reunified to refugees	15 745	141	5.4	7.71 (6.49–9.09)	2.22 (1.82–2.70)	1.67 (1.36–2.05)
Reunified to Danish	59 264	407	5.1	6.86 (6.21–7.56)	1.98 (1.71–2.28)	1.78 (1.52–2.10)
Sex						
Male					Ref	Ref
Female					1.33 (1.17–1.50)	1.10 (0.97–1.25)
Age at study entry					1.01 (1.00–1.01)	1.00 (1.00–1.01)
Substance abuse						
No					Ref	Ref
Yes					1.11 (0.65–1.73)	1.14 (0.68–1.79)
HIV						
No					Ref	Ref
Yes					2.55 (1.09–4.95)	1.06 (0.45–2.07)
Hep C						
No					Ref	Ref
Yes					6.88 (4.10–10.74)	5.90 (3.50–9.23)

a Small numbers suppressed (<5) for data protection reasons.

CI, confidence intervals; Hep B, hepatitis B; Hep C, hepatitis C; HIV, human immunodeficiency virus; *N*, number of participants.

## Discussion

This matched cohort study investigated the incidence of HBV diagnoses among migrants and Danish-born individuals from 1993 to 2015. 179 110 migrants and 1 049 345 Danish-born individuals were included in the cohort, among whom 1362 were registered as diagnosed with HBV within the study period. We found that migrants had a HBV diagnosis rate 30× higher than that among Danish-born individuals, highlighting the importance of developing tailored strategies for at-risk migrant groups in Denmark and similar migrant-receiving countries.

Among the Danish-born cohort, HBV diagnoses were more common among men and those with co-morbidities (HIV, hepatitis C, substance use disorders), consistent with existing research from Denmark [7, 14]. The risk profile among migrants appeared to be different, with female migrants more often diagnosed than males and higher HBV incidence rates associated with region of origin (African and Southeast Asia and Pacific regions) and migrant status (quota refugees and family-reunified migrants). Hospitalization for acute HBV was substantially lower in migrant compared with Danish-born HBV cases, which may reflect the different risk profile and epidemiological pathways—it is likely that migrants are infected with HBV in childhood or through vertical transmission (due to high prevalence in their country of origin) and then proceed to have chronic infection, whereas in Danish populations HBV is mostly contracted in adulthood related to risk factors such as injected drug use. Time from arrival in Denmark to diagnosis also varied by area of origin and migrant status. This highlights the need to ensure that groups who are not included in existing routine screening programs, such as those for pregnant women or in health assessments for quota refugees, do not fall through the gaps.

Many of our findings concur with existing literature on HBV incidence in Denmark and similar migrant-receiving countries [4, 5, 7, 15]. Among migrants, we found high HBV diagnosis rates among those from areas of origin such as the African region and Southeast Asia & Pacific, aligning with HBV prevalence in these areas; the WHO African region and WHO Southeast Asian region together accounted for 85% of all new HBV infections globally in 2022 (63% from WHO African region; 22% WHO Southeast Asian region) [1]. In our cohort, over half (54.4%) of included HBV diagnoses were among women of child-bearing age, and in the migrant cohort, being a woman was associated with higher HBV diagnosis rates. In part, this likely reflects the success of the universal screening program for HBV infection in pregnancy, which has been in place since 2005 in Denmark [7]. Indeed, SSI data in 2023 showed that 56% of women positive for chronic HBV (all of whom were migrants) were found through the antenatal screening program [16], highlighting the importance of effective screening programs to identify infected individuals.

Despite the success of the pregnancy screening program in Denmark and evidence that migrant-born individuals make up the majority of HBV diagnoses and cases in the country, Denmark currently does not meet ECDC guidelines for screening migrants for HBV, which emphasize the importance of HBV screening being offered to all refugees and migrants from intermediate- and high-prevalence countries (≥2% and ≥5% HBsAg positivity, respectively) [6, 17]. Instead, the diagnosis of newly arrived migrants is dependent on either the pregnancy screening programs for women, or several disjointed screening programs based on migration status. For example, since 1989, quota refugees arriving in Denmark routinely receive a pre-arrival General Health Assessment (GHA), including HBV screening, which could explain the high incidence rate (10.18/10 000) and low time to diagnosis (2.4 years) that we identified in this group [10]. In addition, since 1984, Red Cross Denmark has provided asylum seekers with a voluntary GHA conducted at their reception centers, however, this assessment rarely and unsystematically includes screening for hepatitis B [10]. Bollerup *et al.* [7] estimated in 2022 that 7% of HBV cases (around 1000 people) in Denmark are likely to be undiagnosed and only 30% of those diagnosed are linked into care. It is possible that cases diagnosed outside of standard primary care, such as during post-arrival GHAs, may be more challenging to link into further care. Therefore, a more targeted, uniform approach to HBV screening, reporting, and linkage to care in at-risk populations is needed, particularly among those unlikely to be captured by pregnancy screening programs.

### Limitations

This study is a comprehensive and unique analysis of HBV incidence among a large open cohort covering more than 20 years, disaggregated by migrant status. However, several limitations exist that should be considered when interpreting our findings. Firstly, it should be noted that during the study period, there was a change in HBV reporting criteria, with only acute HBV being notifiable before 2000, with this changing to acute and chronic HBV post-2000 [15]. However, as this change in reporting criteria was consistent across the population, this should not have impacted patterns observed between and within cohorts. Further, a recent study has identified some inaccuracy in reporting of HBV cases between different registers in Denmark (e.g. between the National Patient register and the National Disease Surveillance Register) [7]. As this article only drew cases from the Statens Serum Institut’s National Disease Surveillance Register, some HBV cases are likely to have been missed. For hepatitis C, substance use disorders and HBV-related outcomes (e.g. liver cancer), we used diagnoses captured in hospital records, meaning cases treated in primary care or in community settings (or those left untreated) will not be captured. However, this again is unlikely to have had a major impact on the overall patterns we observed in the data.

This study was based on a migrant cohort that included all refugees and family reunited migrants in Denmark, however, the cohort does not include migrants in Denmark on work visas or undocumented migrants, who may differ in their demographics and HBV distribution. Another limitation of this study arises from the way in which the cohorts were created, with Danish-born individuals only included on the date of arrival of their matched migrant. All HBV cases diagnosed before an individual’s entry into the study were excluded, meaning a number of Danish-born cases (*n* = 242) diagnosed between 1993 and individual study entry were excluded, meaning this study is not a full reflection of all Danish HBV cases between 1993 and 2015, but rather a direct comparison between migrant and non-migrant cohorts during the study period.

Finally, while a selection of potential confounding factors were included in this study, further research could be done to explore other factors such as mental health diagnoses influencing hepatitis B diagnoses in these two cohorts.

## Conclusions

In conclusion, our findings highlight the disproportionate burden of HBV in migrants in Denmark, with diagnosis rates influenced by country of origin and migration status. We have also shown that time from arrival to diagnosis varies by migration-related factors, likely influenced by the inconsistency of screening programs available. We suggest that a tailored and consistent approach to screening migrants originating from countries with high prevalences of HBV soon after arrival, particularly those unlikely to be picked up by pregnancy screening programs, will be key to making progress toward the elimination of HBV in Denmark and similar migrant-receiving countries. This approach would bring Denmark into alignment with ECDC guidelines to screen all migrants from intermediate- and high-prevalence countries [17] and with Danish Society for Infectious Medicine (DSI) guidelines for screening at-risk groups in Denmark [18], which evidence suggests are not currently consistently implemented in practice [8].

## Supplementary Material

ckag167_Supplementary_Data

## Data Availability

Further data to support the findings of this study are available on reasonable request from the corresponding author. The complete dataset is not publicly available due to restrictions on access to national registry data for data protection reasons. Key points There is a disproportionate burden (30× higher) of HBV diagnoses among migrants in Denmark compared to Danish-born individuals. Diagnosis rates among migrants are influenced by country of origin and migration status, in contrast to diagnoses among Danish-born individuals, which tend to be correlated with risk factors such as substance use disorders. A tailored and consistent approach to screening migrants originating from countries with high HBV prevalence soon after arrival will be key to making progress toward the WHO’s 2030 HBV elimination goals.
