# Supplementary material for: Persisting disparities in hepatitis B diagnoses, associated risk factors, and time to diagnosis among migrants compared with Danish-born populations: a matched cohort study
Source: Eur J Public Health. 2026 Sep 14;36(5):ckag167. doi: 10.1093/eurpub/ckag167 (PMC13573591; doi:10.1093/eurpub/ckag167)
Supplement: Supplementary file 1 [file ckag167_supplementary_data.zip › ejph-2026-02-om-0101-File007.docx]

**Table S2. Baseline characteristics of the overall cohort**

|  |  | **Danish-born**  **N (%)** | **Migrant**  **N (%)** | **Total n (%)** |
| --- | --- | --- | --- | --- |
| **N** | | 1,049,345 | 179,110 | 1,228,455 |
| **Sex**  Male  Female | | 481,974 (45.9)  567,371 (54.1) | 82,483 (46.1)  96,627 (53.9) | 564,457 (45.9)  663,998 (54.1) |
| **Mean age at study start (SD)** | | 28.3 (13.2) | 28.2 (13.9) | 28.3 (13.3) |
| **Year of Birth**  <1950  1950-1970  1971-1990  >1990 | | 49,451 (4.7)  340,274 (32.4)  518,731 (49.4)  140,889 (13.4) | 10,025 (5.6)  55,968 (31.2)  88,920 (49.6)  24,197 (13.5) | 59,476 (4.8)  396,242 (32.3)  607,651 (49.5)  165,086 (13.4) |
| **Co-morbidities**  HIV+  Substance abuse  Hepatitis C | | 406 (0.03)  31,484 (3.0)  1,241 (0.1) | 538 (0.3)  1,775 (1.0)  346 (0.2) | 944 (0.08)  33,259 (2.7)  1,587 (0.1) |
| **Area of origin**  Central Asia & Eastern Europe  Latin America & Caribbean  Middle East & North Africa  Southeast Asia & Pacific  Stateless  African Region  ‘Western’ countries | |  | 44,602 (24.9)  5,165 (2.9)  50,602 (28.3)  36,468 (20.4)  1,115 (0.6)  25,521 (14.2)  15,637 (8.7) |  |
| **Migrant status**  Asylum-pathway refugee  Quota Refugee  Reunified to immigrants  Reunified to refugees  Reunified to Danish | |  | 83,765 (46.8)  10,082 (5.6)  10,252 (5.7)  15,745 (8.7)  59,264 (33.1) |  |

HIV: Human immunodeficiency virus; N: Number of participants; SD: Standard deviation
