# Supplementary material for: Persisting disparities in hepatitis B diagnoses, associated risk factors, and time to diagnosis among migrants compared with Danish-born populations: a matched cohort study
Source: Eur J Public Health. 2026 Sep 14;36(5):ckag167. doi: 10.1093/eurpub/ckag167 (PMC13573591; doi:10.1093/eurpub/ckag167)
Supplement: Supplementary file 1 [file ckag167_supplementary_data.zip › ejph-2026-02-om-0101-File006.docx]

**Table S1.** Region of origin categorised by included countries

| Region | Countries* |
| --- | --- |
| Eastern Europe and Central Asia | Albania, Belarus, Bosnia and Herzegovina, Cyprus, former Yugoslav Republic, Kazakhstan, Kosovo, Kyrgyzstan, Moldova, Republic of North Macedonia, Russia, Serbia, Tajikistan, Turkmenistan, Turkey, Uzbekistan |
| Western (Europe, North America and Oceania) | Australia, Austria, Belgium, Bulgaria, Croatia, Canada, Czechia, Estonia, Germany, Hungary, Ireland, Italy, Latvia, Lithuania, Malta, Netherlands, New Zealand, Poland, Portugal, Romania, Slovakia, Slovenia, Spain, Switzerland, Ukraine, United Kingdom |
| Latin America and Caribbean | Argentina, Bahamas, Belize, Brazil, Chile, Colombia, Costa Rica, Cuba, Dominican Republic, Ecuador, El Salvador, Guatemala, Guyana, Haiti, Honduras, Paraguay, Peru, Panama, Saint Lucia, Saint Vincent and the Grenadines, Trinidad and Tobago, Uruguay, Venezuela |
| Middle East and North Africa | Algeria, Armenia, Azerbaijan, Bahrain, Djibouti, Egypt, Georgia, Iran, Iraq, Israel, Jordan, Kuwait, Lebanon, Libya, Morocco, Oman, Saudi Arabia, Syria, Tunisia, United Arab Emirates, Yemen |
| South and South-East Asia and Pacific | Afghanistan, Bangladesh, Bhutan, Cambodia, China, Fiji, Hong Kong, India, Indonesia, Japan, Kiribati, Laos, Macao, Malaysia, Maldives, Mongolia, Myanmar/Burma, Nepal, North Korea, Pakistan, Papa New Guinea, Philippines, Samoa, Singapore, Solomon Islands, South Korea, Sri Lanka, Taiwan, Thailand, Tonga, Vietnam |
| Sub-Saharan African | Angola, Benin, Botswana, Burkina Faso, Burundi, Cameroon, Cape Verde, Central African Republic, Chad, Cote D’Ivoire, Democratic Republic of the Congo, Eritrea, Ethiopia, Gabon, Ghana, Guinea, Guinea Bissau, Kenya, Lesotho, Liberia, Madagascar, Malawi, Mali, Mauritania, Mauritius, Mozambique, Namibia, Niger, Nigeria, Rwanda, Senegal, Seychelles, Sierra Leone, Somalia, South Africa, South Sudan, Sudan, Eswatini, Tanzania, The Gambia, Togo, Uganda, Zambia, Zimbabwe |

*Only countries represented in the study cohort are listed.
